# Gene expression changes consistent with neuroAIDS and impaired working memory in HIV-1 transgenic rats

**DOI:** 10.1186/1750-1326-9-26

**Published:** 2014-07-01

**Authors:** Vez Repunte-Canonigo, Celine Lefebvre, Olivier George, Tomoya Kawamura, Marisela Morales, George F Koob, Andrea Califano, Eliezer Masliah, Pietro Paolo Sanna

**Affiliations:** 1 Molecular and Cellular Neuroscience Department, La Jolla, CA 92037, USA; 2 Committee on the Neurobiology of Addictive Disorders, The Scripps Research Institute, La Jolla, CA 92037, USA; 3 INSERM Unit U981, Gustave Roussy Institute, Villejuif, France; 4 National Institute on Drug Abuse, Intramural Research Program, Neuronal Networks Section, Baltimore, MD 21224, USA; 5 National Institute on Alcohol Abuse and Alcoholism, 5635 Fishers Ln, Rockville, MD 20852, USA; 6 Department of Systems Biology, Columbia University, New York, New York 10032, USA; 7 Department of Neuroscience, University of California at San Diego, La Jolla, CA 92093, USA

## Abstract

**Background:**

A thorough investigation of the neurobiology of HIV-induced neuronal dysfunction and its evolving phenotype in the setting of viral suppression has been limited by the lack of validated small animal models to probe the effects of concomitant low level expression of multiple HIV-1 products in disease-relevant cells in the CNS.

**Results:**

We report the results of gene expression profiling of the hippocampus of HIV-1 Tg rats, a rodent model of HIV infection in which multiple HIV-1 proteins are expressed under the control of the viral LTR promoter in disease-relevant cells including microglia and astrocytes. The Gene Set Enrichment Analysis (GSEA) algorithm was used for pathway analysis. Gene expression changes observed are consistent with astrogliosis and microgliosis and include evidence of inflammation and cell proliferation. Among the genes with increased expression in HIV-1 Tg rats was the interferon stimulated gene 15 (ISG-15), which was previously shown to be increased in the cerebrospinal fluid (CSF) of HIV patients and to correlate with neuropsychological impairment and neuropathology, and prostaglandin D2 (PGD2) synthase (Ptgds), which has been associated with immune activation and the induction of astrogliosis and microgliosis. GSEA-based pathway analysis highlighted a broad dysregulation of genes involved in neuronal trophism and neurodegenerative disorders. Among the latter are genesets associated with Huntington’s disease, Parkinson’s disease, mitochondrial, peroxisome function, and synaptic trophism and plasticity, such as IGF, ErbB and netrin signaling and the PI3K signal transduction pathway, a mediator of neural plasticity and of a vast array of trophic signals. Additionally, gene expression analyses also show altered lipid metabolism and peroxisomes dysfunction. Supporting the functional significance of these gene expression alterations, HIV-1 Tg rats showed working memory impairments in spontaneous alternation behavior in the T-Maze, a paradigm sensitive to prefrontal cortex and hippocampal function.

**Conclusions:**

Altogether, differentially regulated genes and pathway analysis identify specific pathways that can be targeted therapeutically to increase trophic support, e.g. IGF, ErbB and netrin signaling, and reduce neuroinflammation, e.g. PGD2 synthesis, which may be beneficial in the treatment of chronic forms of HIV-associated neurocognitive disorders in the setting of viral suppression.

## Background

HIV-associated dementia, opportunistic infections and neoplasms are significantly reduced since the introduction of combination antiretroviral therapy (cART) [1-4]. However, decreased HIV-associated dementia has been associated with an increased prevalence of milder and chronic forms of HIV-associated neurocognitive disorders (HAND) and HIV-associated major depressive disorder along with increased life expectancy [4-10].

Release of HIV proteins and cytokine/chemokines from monocytes/macrophages into the CNS parenchyma plays a central role in HIV-associated neurological disorders [11,12]. Several HIV-1 proteins also possess neurotoxic potential including gp41, Vpr, Nef, Rev and Vpu [13-16]. Evidence suggests that the toxic actions of low levels of HIV-1 products are key in the neuropathogenesis of persistent central nervous system HIV disease in the setting of cART. In fact, after the introduction of cART, HAND do not correlate with indicators of florid HIV replication such as plasma viral load and low CD4^+^ counts [4], but are correlated with synaptodendritic injury [17,18], which in experimental settings can be induced by HIV-1 products even in the absence of virus replication [19-21]. Therefore, there is a need to investigate the pathologic consequences of the expression of low levels of multiple HIV-1 proteins in disease relevant cell types.

The HIV-1 transgenic (Tg) rats used in the present study harbor a gag/pol-deleted HIV-1 provirus under the LTR promoter [22], resulting in the co-expression of multiple HIV-1 proteins in disease-relevant central nervous system (CNS) cells such as microglia and astrocytes, but not in neurons [22,23]. Thus, the HIV-1 Tg rats differ from most other non-replicating small animal models expressing a single HIV-1 protein and from replicating models [24]. The construct used in HIV-1 Tg rats was previously used in mice where it was characterized by ectopic expression [25], possibly because of deficient interaction of Tat with the murine cyclin T [26]. Despite lack of virus replication and production, HIV-1 Tg rats have a progressive clinical presentation leading to a picture reminiscent of some of AIDS features later in life that includes immunological abnormalities and neurological manifestations [22,27].

Here we studied global gene expression in the hippocampus of HIV-1 Tg rats before the appearance of overt symptomology to model the effects of concomitant expression of low levels of multiple HIV-1 products in the CNS in the absence of viral replication, as seen in the context of viral suppression in the setting of combination antiretroviral therapy (cART). Results show that HIV-1 Tg rats have gene expression changes reminiscent of neuroAIDS, significant astrogliosis and microgliosis, and working memory impairments. We used the Gene Set Enrichment Analysis (GSEA) algorithm [28] to identify molecular pathways that are differentially activated in HIV-1 Tg rats. We observed dysregulation of pathways associated with gliosis, consistent with the morphological results in HIV-1 Tg rats, reduced trophic support and synaptic plasticity, mitochondrial function, as well as neurodegenerative diseases such as Huntington’s disease and Parkinson’s disease. HIV-1 Tg rats showed working memory impairments in spontaneous alternation behavior in the T-Maze, a paradigm sensitive to prefrontal cortex and hippocampal function, supporting the functional significance of the gene expression changes observed.

## Results

### Astrogliosis and microgliosis in HIV-1 Tg rats

**Figure 1:**
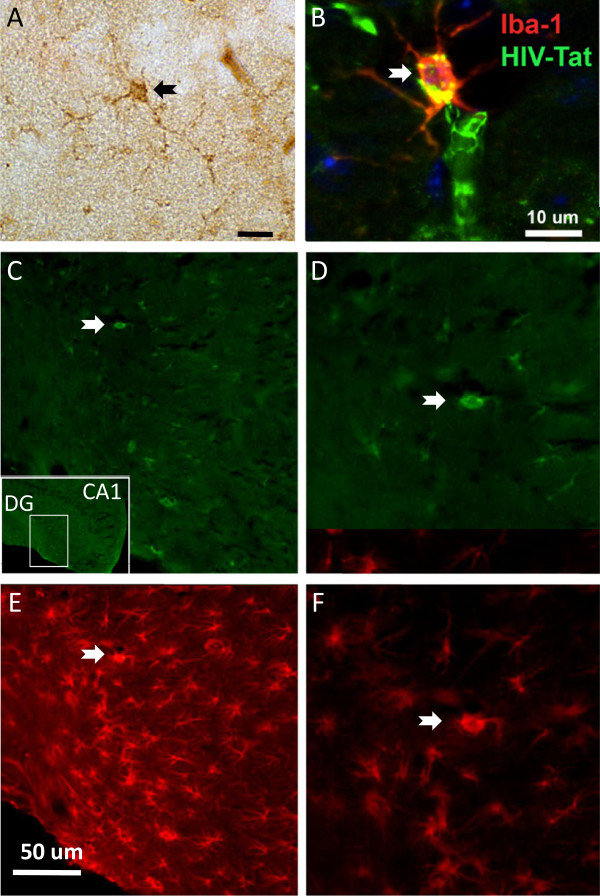
**Expression of HIV-1 products in HIV-1 Tg rats. A)** HIV protein Tat was detected by immunohistochemistry with peroxydase detection in the hippocampus in cells with microglia morphology (arrow) and **B)** by immunofluorescence with double labeling of HIV-1 Tat and the microglia marker Ionized calcium binding adaptor molecule 1 (Iba-1) (arrow). **C-F)** Double immunofluorescence for HIV-1 gp120 **(C-D)** and for the astrocytic marker glial fibrillary acidic protein (GFAP) **(E-F)** in the hippocampus of HIV-1 Tg rats revealed gp120-immunoreactive astrocytes (arrow). The location of the field shown in **C-F** is indicated by the inset in **C**.

**Figure 2:**
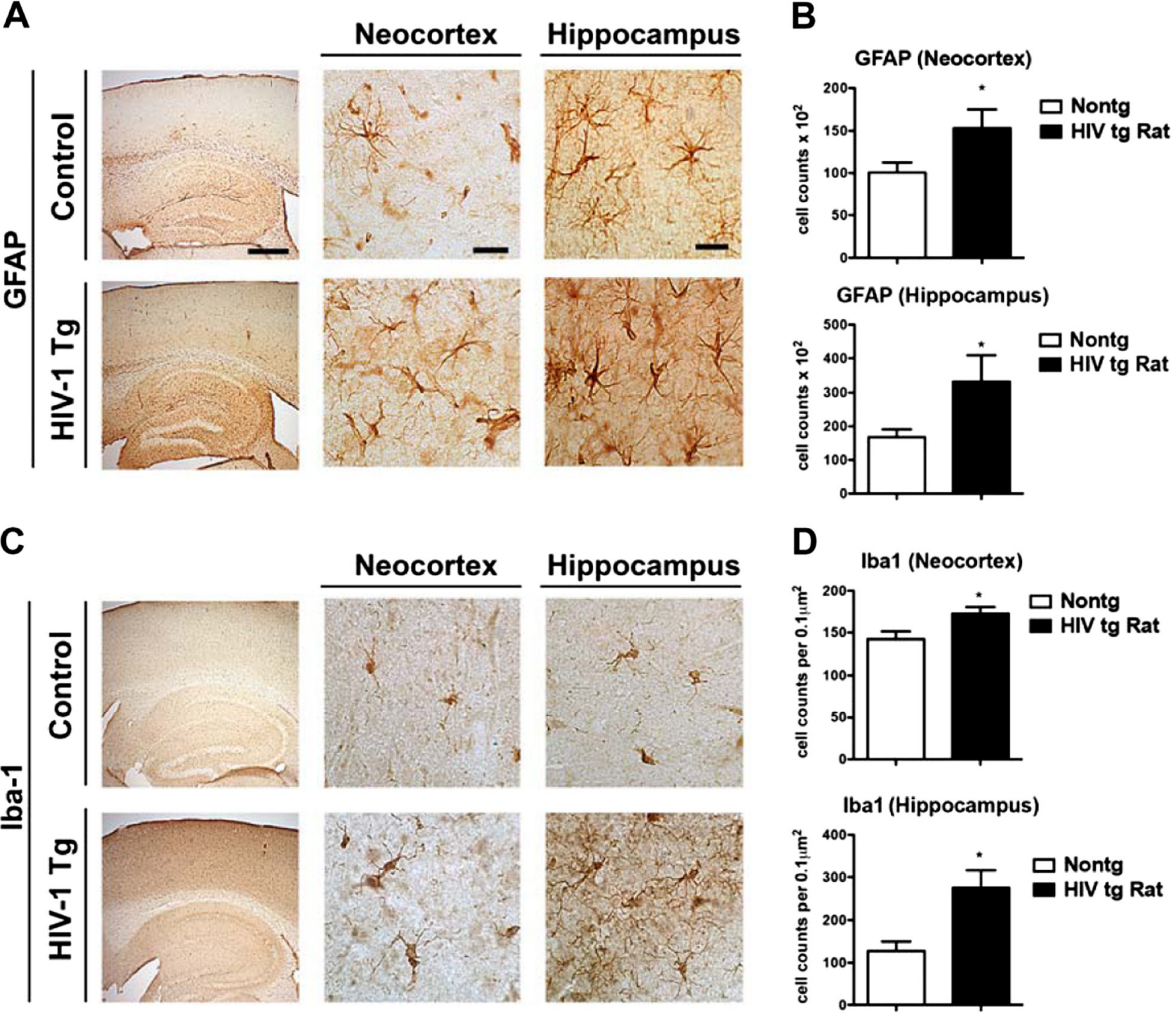
**Astrogliosis and microgliosis in the brain of HIV-1 Tg rats. A)** Immunostaining for the astrocytic marker GFAP in HIV-1 Tg and control rats. The panel to the left is a low power view (4X) of the neocortex and hippocampus, while the panels in the middle and right are at higher magnification (400X) **(B)** Computer aided image analysis of the numbers of GFAP positive cells in the neocortex and hippocampus demonstrate a significant increase in astroglial cells in the HIV-1 tg rats (Student’s *t-*test). **C)** Immunostaining for the microglial marker Iba-1 in HIV-1 Tg and control rats. The panel to the left is a low power view (4X) of the neocortex and hippocampus, while the panels in the middle and right are at higher magnification (400X); **D)** Computer aided image analysis of the numbers of Iba-1 positive cells in the neocortex and hippocampus demonstrate a significant increase in astroglial cells in the HIV-1 Tg rats (* = p < 0.01; n = 6, by *t*-test). Bar = 250 and 25 μm at 4X and 400x, respectively.

**Figure 3:**
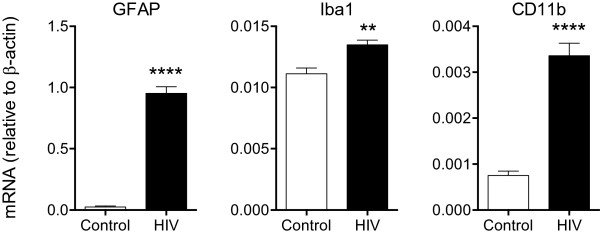
**RT-PCR validation of increased expression of astrogliosis and microgliosis markers in the hippocampus of HIV-1 Tg rats.** The mRNA for the astrocytic marker GFAP, and the microglia markers Iba-1 and CD11b (Mac1) were significantly increased in HIV-1 Tg rats over controls, consistent with the immunohistochemistry results in Figure 2 (*n* = 6, **p < 0.01, ****p < 0.0001, by *t*-test).

Characterization of the neuropathological alterations in the HIV-1 Tg rat was performed with antibodies against the astroglial marker GFAP and the microglial cell marker Iba1. Expression of HIV-1 proteins in the hippocampi of HIV-1 Tg rats was observed in both microglia and astrocytes (Figure 1). As expected in the control animals, scattered astroglial cells were detected in the neocortex and more active astroglia was observed in the adjacent white matter and hippocampus (Figures 1 and 2). In contrast, in HIV-1 Tg rats, levels of astrogliosis in the neocortex and hippocampus were significantly increased with the presence of enlarged astroglia with distended cytoplasm (Figure 2A). Likewise, in control rats, moderate numbers of microglial cells were detected in the neocortex and hippocampus, in contrast in the HIV-1 Tg animals there was an increase in Iba1 positive microglial cells that was more prominent in the hippocampus than in the neocortex (Figure 2C). These cells displayed abundant branches and processes and the cytoplasm was distended, suggesting the possibility of an activated state. Significant astrogliosis and microgliosis were observed by immunostaining in the hippocampi of HIV-1 Tg rats (Figure 2B, D). Quantitative reverse transcriptase polymerase chain reaction (RT-PCR) confirmed that the expression of astrogliosis and microgliosis markers was increased in the hippocampus of HIV-1 Tg rats (Figure 3).

### Gene expression changes in HIV-1 Tg rats

**Figure 4:**
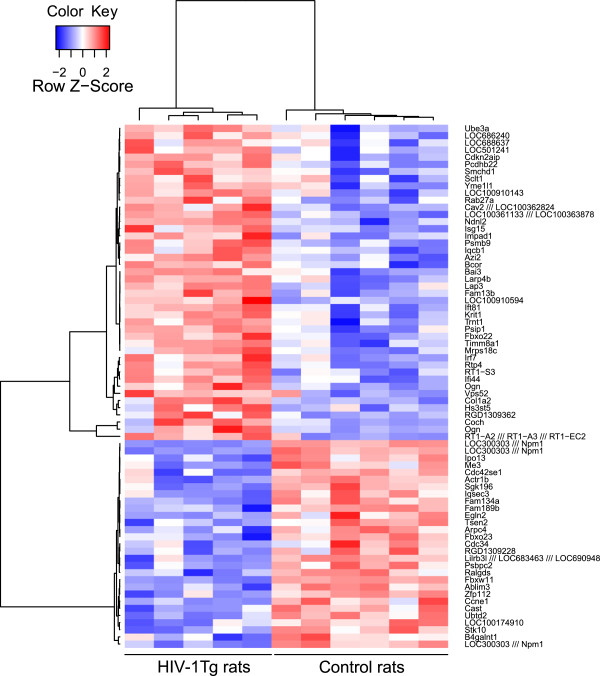
**Hierarchical clustering of top differentially expressed genes in the hippocampus of HIV-1 Tg rats (HIV) versus wild-type littermate controls (Control).** Gene expression was profiled in the hippocampi of HIV-1 and wild-type rats with high-density Affymetrix microarrays. The dendrogram shows 73 of the top 100 differentially expressed probesets that were found to be associated with gene names (Additional file 3: Table S3). Genes are colored according to their expression values. Red indicates upregulated genes in HIV-1 Tg rats, while blue indicates downregulated genes. Brightness is proportional to the extent of change in gene expression.

**Figure 5:**
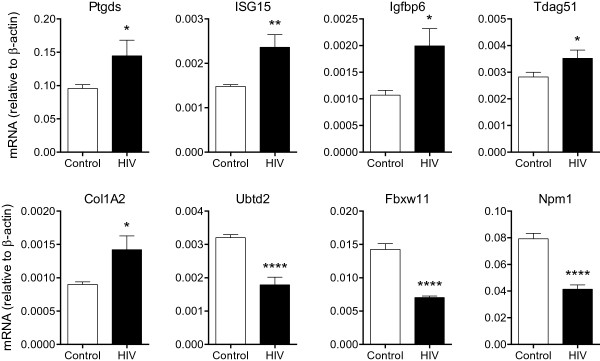
**RT-PCR validation of differentially expressed genes in the hippocampus of HIV-1 Tg rats.** Examples of genes that were found to be differentially regulated in the hippocamous of HIV-1 Tg rats include PGD2 synthase (Ptgds), interferon stimulated gene ISG-15 (ISG-15), IGF binding protein 6 (Igfbp6) and the IGF-activated gene T cell death-associated gene 51 (TDAG51), Col1A2 (procollagen type1-a2), and the mitochondrial protein ubiquitin domain containing 2 (Ubtd2). Lastly, among differentially expressed genes are Fbxw11 and Npm1, which interact with HIV-1 proteins Vpu and Tat, respectively. Please see text (*n* = 6, *p < 0.05, **p < 0.01, ***p < 0.001, ****p < 0.0001, by *t*-test).

**Figure 6:**
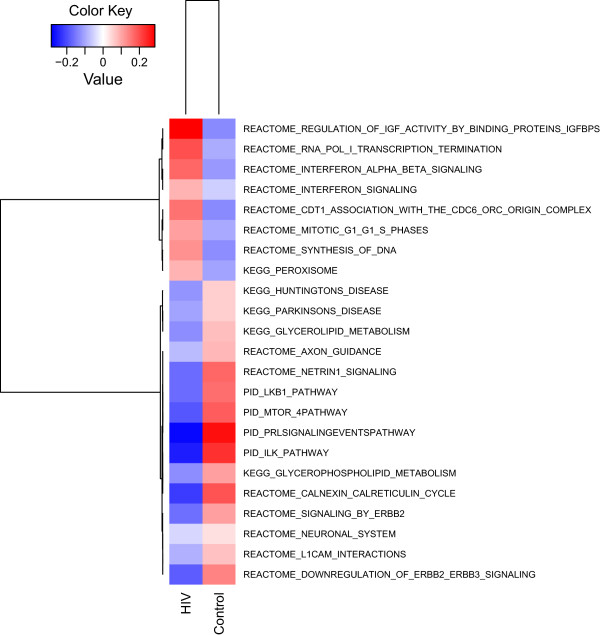
**Top 23 differentially regulated pathways in the hippocampus of HIV-1 Tg rats and controls by GSEA.** The Gene Set Enrichment Analysis (GSEA) algorithm was used for pathway analysis using the MSigDB C2 canonical pathway collection [28]. Of the significantly differentially regulated pathways with statistical significance of p ≤ 0.01, 8 showed increased activation, while the rest was decreased. Among the increased ones are pathways consistent with astrocyte and microglia activation, inflammatory processes, and interferon activation. Decreased pathways indicate substantial downregulation of signaling systems involved in neuronal trophism and synaptic synaptic function. Pathways are colored according to their expression values: red indicates upregulated genes in HIV-1 Tg rats, while blue indicates downregulated pathways. Brightness is proportional to the extent of change in pathway expression.

**Figure 7:**
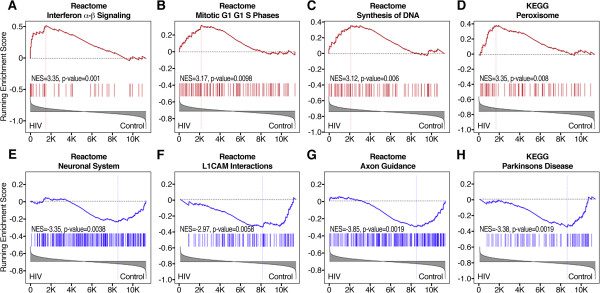
**GSEA plots of selected differentially regulated pathways.** Representative differentially regulated pathways are shown including pathways with increased and reduced activation in the hippocampus of HIV-1 Tg rats. Pathways with increased activation included pathways indicative of **(A)** interferon activation; **(B, C)** cell division, consistent with astrogliosis and microgliosis; **(D)** peroxisome dysregulation. Pathways with reduced activation **(E-H)** are indicative of reduced neuronal trophism and neurodegeneration.

Gene expression was profiled in the hippocampi of HIV-1 Tg and wild-type rats with high-density Affymetrix arrays (Figures 4 and 5, Additional files 1 and 2: Tables S1 and S2). Pathway analysis with the GSEA algorithm [28] revealed 23 pathways significantly differentially regulated with statistical significance of p ≤ 0.01 (Figures 6 and 7, Additional file 3: Table S3). Eight differentially regulated pathways showed increased activation, while the rest was decreased.

Results show that among the pathways and genes differentially expressed, several are consistent with astrocyte and microglia activation and inflammatory processes (Figures 2 and 3, Additional file 3: Table S3). In particular, pathways showing significant activation included 4 pathways indicative of DNA synthesis and cell proliferation, suggestive of astrocyte and microglia activation (“REACTOME_SYNTHESIS_OF_DNA” and “REACTOME_MITOTIC_G1_G1_S_PHASES”, “REACTOME_CDT1_ASSOCIATION_WITH_THE_CDC6_ORC_ORIGIN_COMPLEX” and “REACTOME_RNA_POL_I_TRANSCRIPTION_TERMINATION”, MSigDB geneset denomination; Figures 4 and 7, Additional file 3: Table S3). Two pathways indicative of interferon activation were also increased (“REACTOME_INTERFERON_SIGNALING” and “REACTOME_INTERFERON_ALPHA_BETA_SIGNALING”) (Figures 5 and 6, Additional file 3: Table S3). Among the interferon-related genes that were found to be increased in HIV-1 Tg rats is the interferon stimulated gene 15 (ISG-15) (Figure 5, Additional file 1: Table S1). Other inflammation- and astrogliosis-related genes increased in the hippocampi of HIV-1 Tg rats include prostaglandin D2 (PGD2) synthase (Ptgds) and procollagen type1-a2 (Col1A2) (Figure 5, Additional file 1: Tables 1).

The GSEA analysis highlights a substantial downregulation of intracellular signaling pathways relevant to trophic support. In particular, the PI3K-mTOR signaling pathway (“PID_MTOR_4PATHWAY”), the LKB1 pathway (“PID_LKB1_PATHWAY”), and the integrin-linked kinase (ILK) pathway (“PID_ILK_PATHWAY”), were downregulated (Figures 6 and 7, Additional file 3: Table S3). Various downregulated genesets also contain numerous MAP kinases (PID_MTOR_4PATHWAY; REACTOME_AXON_GUIDANCE; PID_PRLSIGNALINGEVENTSPATHWAY; REACTOME_L1CAM_INTERACTIONS; REACTOME_SIGNALING_BY_ERBB2; REACTOME_DOWNREGULATION_OF_ERBB2_ERBB3_SIGNALING”, “PID_ILK_PATHWAY”), such as ERK1 (MAPK3).

GSEA also showed downregulation of genesets related to neuronal function and synaptic plasticity such as “REACTOME_NEURONAL_SYSTEM” and “REACTOME_AXON_GUIDANCE” (Figures 6 and 7, Additional file 3: Table S3), which appears consistent with the reduced trophic tone suggested by the data.

GSEA pathway analysis also indicates that key roles in the downregulation of transduction pathways mediating trophic signals may be played by the insulin-like growth factor (IGF), Erb-neuregulin-1 (NRG1) and netrin systems. In particular, IGF binding proteins 2 and 6 (Igfbp2 and Igfbp6) and the IGF-activated gene T cell death-associated gene 51 (TDAG51), also known as pleckstrin homology-like domain family A member 1 (PHLDA), were differentially regulated in HIV-1 Tg rats (Figure 5, Additional file 1: Table S1) as was the IGF signaling pathways by GSEA (REACTOME_REGULATION_OF_INSULIN_LIKE_GROWTH_FACTOR_IGF_ACTIVITY_BY_INSULIN_LIKE_GROWTH_FACTOR_BINDING_PROTEINS_IGFBPS). GSEA also showed differential regulation of Erb-neuregulin-NRG1 and netrin signaling pathways (“REACTOME_SIGNALING_BY_ERBB2”, “REACTOME_DOWNREGULATION_OF_ERBB2_ERBB3_SIGNALING”, and “REACTOME_NETRIN1_SIGNALING”; Figures 6 and 7, Additional file 3: Table S3).

Pathways related to neurodegeneration associated with protein misfolding and mitochondrial dysfunction were differentially regulated including “KEGG_PARKINSONS_DISEASE” and “KEGG_HUNTINGTONS_DISEASE”. Another significantly dysregulated pathway is the “REACTOME_CALNEXIN_CALRETICULIN_CYCLE”, which involves unfolded protein response genes.

Multiple genesets are indicative of dysregulation of phospholipid metabolism (“EGG_GLYCEROPHOSPHOLIPID_METABOLISM”, “KEGG_GLYCEROLIPID_METABOLISM” and “KEGG_PEROXISOME”; Figures 6 and 7, Additional file 3: Table S3).

Lastly, two cellular targets of HIV-1 were differentially regulated in the hippocampus of HIV-1 Tg rats, Fbxw11 and the nuclear chaperone nucleophosmin/B23 encoded by the Npm1 gene (Figure 5 and Additional file 1: Table S1).

### Working memory impairments in HIV-1 Tg rats

**Figure 8:**
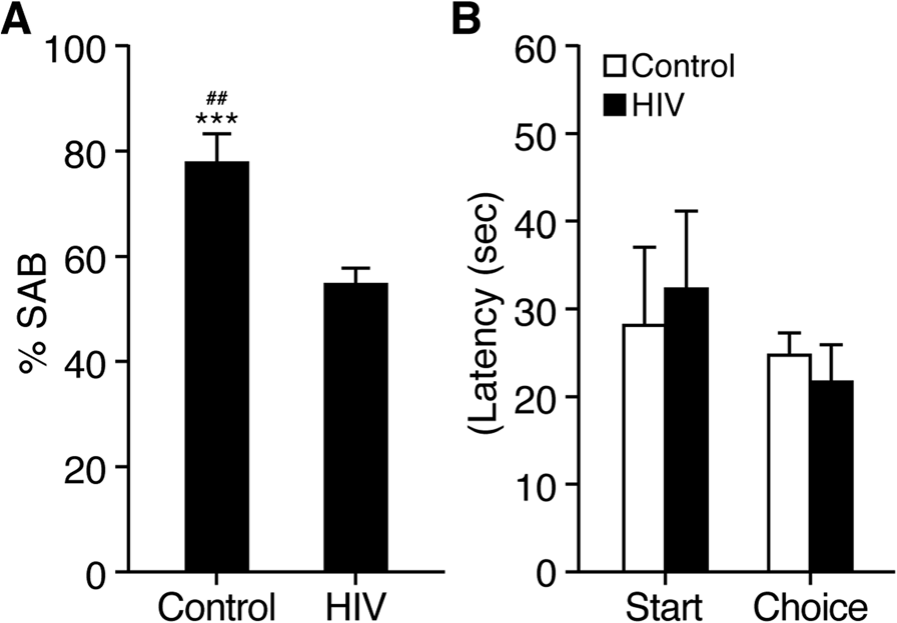
**Working memory impairment in HIV-1 Tg rats.** Working memory was significantly impaired in adult (4–5 months old) HIV-1 Tg rats compared to wild-type controls (CONT) using the *spontaneous alternation behavior in the T-Maze*, a paradigm sensitive to prefrontal cortex and hippocampal impairments. **A)** HIV-1 Tg rats exhibited a marked decrease of spontaneous alternation behavior (SAB), a measure of working memory, compared to control rats (n = 7 for both groups; t10 = 4.2, p < 0.01). Only the control rats alternated significantly above chance (t6 = 7.1, p < 0.001) whereas HIV-1 Tg rats were not significantly different from chance level (t4 = 1.2, p = ns). There was no difference between the two groups in the latency to leave the start box or the latency to make a choice **(B)**. ***p < 0.001 *vs.* chance; p < 0.01 *vs.* HIV-1 Tg rats.

To determine the functional correlates of the present gene expression and pathologic findings, we then investigated working memory in HIV-1 Tg rats using a hippocampus and prefrontal cortex-dependent task [29-32]. Rats were tested in the dark in spontaneous alternations in the T-Maze, which is a paradigm sensitive to prefrontal cortex and hippocampal impairments [29-32]. Visual cues were not provided in the T-Maze paradigm, as done by others [33], to minimize the influence of cataracts in HIV-1 Tg rats. HIV-1 Tg rats exhibited a marked decrease of alternation behavior (SAB) compared to control rats (t_10_ = 4.2, p < 0.01) (Figure 8A). Only the control rats alternated significantly above chance (t_6_ = 7.1, p < 0.001) whereas HIV-1 Tg rats were not significantly different from chance level (t_4_ = 1.2, p = ns). There was no difference between the two groups in the latency to leave the start box or the latency to make a choice (Figure 8B). Finally there was no difference between the two groups in the number of failed trials or in the response bias (Control: 0.55 ± 0.02; HIV-1 Tg: 0.58 ± 0.04, p = ns). These results indicate an impairment of hippocampal working memory in HIV-1 Tg rats that was not a consequence of an increase of anxiety-related behavior since we did not see increases in the latency to leave the start box or in the number of failed trials in HIV-1 Tg rats. Moreover this alteration of working memory is unlikely to come from an increase in the delay between the encoding and the recall of information or an alteration of locomotor activity since the latency to start and the latency to enter the arm were similar in both groups. Thus, the gene expression changes observed above are associated with neurocognitive impairments in HIV-1 Tg rats.

## Discussion

### HIV-1 Tg rats as a model of neuroAIDS

A thorough neurobiological investigation of the neurobiology of HIV-induced neuronal dysfunction and its evolving phenotype in the context of viral suppression has been limited by the lack of validated small animal models to probe the effects of concomitant low level expression of multiple HIV-1 products in disease-relevant cells in the CNS. The HIV-1 Tg rats are constitutive, making them a model of persistent exposure to viral proteins, although lack of induciblity of the HIV-1 provirus can be seen as a limitation. Here we show that HIV-1 Tg rats express viral proteins in microglia and astrocytes, display astrogliosis and microgliosis, and have gene expression changes consistent with human neuroAIDS. Collectively, these results support that HIV-1 Tg rats are a valid animal model of neuroAIDS.

Supporting the functional significance of the gene expression alterations reported here, HIV-1 Tg rats showed working memory impairments in spontaneous alternation behavior (SAB) in the T-Maze, a paradigm sensitive to prefrontal cortex and hippocampal function. Rats were tested in the dark in the T-Maze with no visual cues provided to minimize the influence of cataracts in HIV-1 Tg rats, as done by others [33]. In this regard it should be noted that spontaneous alternation behavior (SAB) has been used in aged rats with visual impairments [34] and to compare anophthalmic mutant mice to wild-type controls [35]. It has been previously shown that HIV transgenic rats exhibit impairment in a spatial reversal learning task [36] without any deficit in spatial memory or motor activity [37]. Our results extend these studies by showing that HIV rats also exhibit impairment in a working memory task. The deficits in the reversal learning task and the working memory task may both be explained by an impairment in the ability of HIV rats to shift attention to a new target, a decreased working memory capacity and/or an increase in perseveration.

### Gene expression changes in HIV-1 Tg rats

Gene expression and pathway analysis with the GSEA algorithm show evidence of neuroinflammatory processes and gliosis and synaptodendritic injury that together with the impairment in working memory are reminiscent of HAND in humans. In particular, the GSEA-based pathway analysis that we employed suggests key dysregulation in IFN regulated pathways, including ISG-15, which exerts antiviral activities through members of the Endosomal sorting complex required for transport (ESCRT) proteins [38]. ISG-15 was proposed to be a predictive biomarker for HAND [39,40]. In particular, ISG-15 was shown to correlate with neuropathology and viral load and ISG-15 CSF levels may be predictive of future neurocognitive impairments [38]. Several other inflammation-related genes induced in the hippocampus of HIV-1 Tg rats were previously associated with neuroAIDS in humans including IGF binding proteins [41], prostaglandin D2 (PGD2) synthase [42], and the pro-alpha2 chain of type I collagen, Col1A2 [43]. Prostaglandins have been implicated in inflammation-induced working memory deficits [44] and imaging evidence supports that brain arachidonic acid metabolism in HIV-1 transgenic rats is upregulated [45]. PGD2 has been implicated in the induction of astrogliosis and demyelination [42,46] and has been associated with immune activation, astrogliosis, microgliosis, and neuroAIDS in humans [42]. Col1A2 has been associated with neuroAIDS in humans [43]. Collagen induction in the CNS has been also observed in brain injury, Alzheimer’s disease, temporal lobe epilepsy, and multiple sclerosis plaques [47-49].

GSEA analysis showed downregulation of genesets related to neuronal function, neuronal trophism, synaptic plasticity, as well as genes and pathways associated with degenerative diseases such as Huntington’s disease and Parkinson’s disease, which include genes involved in protein misfolding and mitochondrial function. Decreased expression of synaptic plasticity and axon guidance genes was also observed in patients with HIVE in the The National NeuroAIDS Tissue Consortium Brain Gene Array [50] and in another study [43], consistent with the notion that HAND in the cART setting correlates with synaptodendritic injury [17,18]. Dysregulations of genes related to Parkinson’s disease and Huntington’s disease and mitochondrial function, is consistent with previous studies suggesting mitochondrial dysfunction and oxidative stress as potential contributors to HAND [51].

Signaling systems involved in neuronal trophism and synaptic maturation and plasticity that were found to be differentially regulated in HIV-1 Tg rats involve IGF, ErbB and netrin, all of which have been shown to exert trophic actions on dendritic spines [52-55], suggesting that dysregulation of these pathways can contribute to the synaptodendritic injury seen in neuroAIDS [17,18].

IGF signaling has been implicated in synaptic trophism, depression and Parkinson’s disease [55-57]. GSEA highlighted differential expression of IGF signaling genes including increased Igfbp2 and Igfbp6 expression, which were also found to be increased in the CSF of HIV-1 patients [41]. The IGFBPs have high affinities for the IGFs [58,59] and their increased expression may result in reduced trophic support as well as inflammatory processes [60-62]. The IGF activated gene TDAG51/PHLDA1 [63], which is expressed in both neurons and glial cells [64], is implicated in insulin signaling [65] and has been shown to have differential effects on susceptibility to apoptosis [66-68].

ErbB2/B4 receptors and their ligand neuregulin-1 (NRG1) are encoded by candidate susceptibility genes for schizophrenia [69]. Functional NRG1 receptors consist of ErbB4 homodimers or heterodimers between ErbB2, ErbB3 and ErbB4 since NRG1 binds ErbB3 and ErbB4, and ErbB2 and ErbB4 have intrinsic tyrosine kinase activity [69]. Mice that lack the ErbBs with intrinsic tyrosine kinase activity (ErbB2 and ErbB4) in the CNS have reduced dendritic spine density and behavioral abnormalities [53], possibly implicating this ErbB signaling in synaptodendritic injury. Netrins are secreted mediators that exert multiple trophic actions including axon guidance [70], synaptic plasticity [71], prevention of apoptosis [72,73], adult neurogenesis including in the course of CNS regeneration [74-76], which is believed to be impaired in neuroAIDS [77], and synaptic trophism [54].

The GSEA analysis highlights a substantial downregulation of intracellular signaling pathways relevant to trophic support such as the PI3K-mTOR signaling pathway, which mediates a vast array of trophic signals [78]; the LKB1 pathway, which is key to neuronal survival following mitochondrial insults [79] and cross-talks with the PI3K-mTOR pathway; and the ILK pathway, which is involved in mediating trophic signals of the extracellular matrix and trophic factors as well as in the anti-apoptotic effect of the PI3K pathway [80]. Recent studies have indicated impaired processing and transport of neurotrophic factors in HIV-1 neuropathogenesis [81-83]. The PI3K-mTOR signaling pathway is involved in synaptic plasticity [84-86] and was shown to be dysregulated in the frontal cortex of patients with neuroAIDS [43]. Downregulated genesets also contain numerous MAP kinases including ERK1 (MAPK3), which was previously implicated in the neurological actions of HIV-1 products [87].

The GSEA analysis also points to coordinated mitochondria and peroxisome dysregulations as well as dysregulation of phospholipid metabolism. Peroxisomes and mitochondria exhibit a functional interplay in fatty acid processing and intermediate metabolism [88,89]. It could be envisioned that if peroxisomal metabolism is slowed, critical metabolic intermediates (e.g., acetyl-CoA) may not be adequately supplied to mitochondria and, conversely, disruption of mitochondrial metabolism can similarly affect peroxisomal function. Peroxisome dysfunction may also contribute to increased lipid peroxidation and cellular aging in HIV-1 [90,91]. In apparent agreement with the dysregulation of these pathways, evidence suggestive of altered phospholipid metabolism has been reported in HIV-1 patients [92,93] and peroxisome dysregulation can be indicative of altered brain oxidative balance associated with HIV-1 [94].

Two cellular targets of HIV-1, Fbxw11 and nucleophosmin/B23, were also differentially regulated in the hippocampus of HIV-1 Tg rats. Fbxw11 is the gene coding for βTrCP, a cellular ubiquitin ligase that was found to be bound by the HIV-1 Vpu viral protein [95]. Nucleophosmin/B23, encoded by the Npm1 gene, is a nuclear chaperone implicated in the nuclear transport of Tat [96].

### Potential therapeutic targets for neuroAIDS suggested by gene expression analysis of HIV-1 Tg rats

Gene expression analysis can be a useful tool both to gain insights in the pathogenesis and for the identification of potential new therapeutic targets by identifying the signaling pathways that have the potential to modify the disease pathophysiology [97-99]. This is especially needed in neuroAIDS as no targeted therapy other than cART is currently recommended for the management of HAND [100,101]. Adjunctive therapies for HAND explored to date include the low-affinity antagonist of the NMDA type glutamate receptor memantine, the calcium channel blocker nimodipine, and the monoamine oxidase B inhibitor selegiline, in addition to antioxidants and anti-inflammatory drugs such as minocycline. Memantine and nimodipine are intended to protect from excitotoxic neuronal damage associated with excessive glutamate release [102,103]. Selegiline is a drug used for the treatment of early-stage Parkinson’s disease, depression and dementia [104,105].

Our gene expression results showing decreased expression of Parkinson's disease-related genesets lend support for the use of selegiline in HAND and potentially for other therapeutics used in Parkinson's disease such as methylphenidate, a stimulant that reduces reuptake of dopamine and norepinephrine that has proven beneficial in lowering fatigue scores in HIV patients [106]. The present results of increased PGD2 synthase (Ptgds), is in keeping with previous studies that showed elevated PGD2 levels in HIV-1-positive patients [42]. A potential role of prostaglandins in HIV-1 neuropathogenesis is also indicated by increased expression of the prostaglandin synthetic enzyme COX-2, which is also a characteristic of other degenerative conditions such as Alzheimer's disease and amyotrophic lateral sclerosis [107,108]. Dysregulation of prostaglandin synthesis in HAND lends support to the use of inhibitors of cyclooxygenase-2 (COX-2) as well as newer compounds targeting the PGD2 receptor. In this regard, a COX-2 inhibitor has shown promise in downregulating immune activation and improving T cell function in HIV-1 patients [109].

The present gene expression results as well as the previous literature also point to a role in HAND of several signaling systems with the potential to provide trophic support to synapses and reverse the synaptodendritic injury associated with HAND [17,18]. Among them are IGF, ErbB and netrin signaling and the PI3K-mTOR signal transduction pathway, which mediates trophic and plastic actions of various signaling systems.

IGF-1 therapy has shown potential in models of both Parkinson’s disease [110,111] and Alzheimer's disease [112,113]. These effects are at least in part mediated by recruitment of PI3K [111,114]. Additionally, chronic treatment with IGF-1 was protective *in vitro* against gp120-mediated neuronal damage and was synergistic with erythropoietin (EPO), at least in part, through cooperative activation of PI3K [115]. GLP-1 and GLP-1 analogs, which activate partially overlapping signal transduction pathways as insulin and IGF-1 [114], have also shown beneficial effects in preclinical models of neurodegenerative disorders [116-119]. There is currently great interest in developing drugs to modulate NRG1**–**ErbB4 and netrin signalling [120,121]. Netrin agonists may be potentially beneficial in neuroAIDS both by exerting neurotrophic actions and by reducing inflammation [120]. The PI3K pathway is itself a potential target for reversal of the synaptodendritic injury associated with HAND. However, as PI3K appears to play a role in the regulation of the HIV-1 LTR promoter and virus latency [122,123], activation of PI3K in the cells that harbor the provirus may increase levels of expression of HIV-1 products, potentially leading to detrimental effects on cognition.

## Conclusions

The investigation of HIV-induced CNS dysfunction in the context of viral suppression has been limited by the lack of validated small animal models with low level expression of multiple HIV-1 proteins in disease-relevant cells. Here we show that HIV-1 Tg rats express viral proteins in microglia and astrocytes and display astrogliosis and microgliosis and show gene expression changes that are reminiscent of HIV-1 infection in humans. Differentially regulated genes and pathway analysis also identify specific pathways that can be targeted therapeutically to increase trophic support and reduce neuroinflammation that may be beneficial in the treatment of chronic forms of HAND in the setting of cART.

## Methods

### Animals

HIV-1 Tg and wild-type rats 4–5 months of age (Harlan Sprague–Dawley) were used for the study. For immunohistochemistry animals were perfused under deep isofluorane anesthesia with 4% paraformaldehyde in phosphate buffer; for microarray analyses, animals were sacrificed under deep isofluorane anesthesia and hippocampi were quickly removed and processed for RNA extraction.

### Immunocytochemical analysis and image analysis

Brains were post-fixed in phosphate-buffered 4% paraformaldehyde at 4°C for 48 hrs and sectioned in the saggital plane at 40 μm with a Vibratome 2000 (Leica). Briefly as previously described [124] saggital brain vibratome sections from the non-Tg and HIV-1 Tg rats were incubated overnight at 4°C with the rabbit polyclonal antibodies against Tat (NIH AIDS reagent program; cat# 1974) or mouse monoclonal antibodies against the astroglial marker - glial fibrillary acidic protein (GFAP, Millipore), or a monoclonal antibody against the microglial marker Iba-1 (1:1000, Sigma-Aldrich, Saint Louis, MO). Primary antibody incubation was followed by incubation with secondary biotinylated IgG, then avidin-HRP and diaminobenzidine (DAB) detection. All sections were processed under the same standardized conditions. Immunostained sections were imaged with a digital Olympus microscope and assessment of levels of Tat, GFAP and Iba-1 immunoreactivity was performed utilizing the Image-Pro Plus program (Media Cybernetics, Silver Spring, MD). For each case a total of three sections (10 digital images per section at 400x) were analyzed in order to estimate the average number of immunolabeled cells per unit area (mm2) and the average intensity of the immunostaining (corrected optical density). All slides were processed simultaneously under the same conditions and experiments were performed in triplicate to assess the reproducibility of results. To determine the co-localization between HIV-1 proteins and astroglial and microglial cells, double-labeling experiments were performed, as previously described. For this purpose, vibratome sections were immunolabeled with the rabbit polyclonal antibody against human Tat or gp120 (NIH AIDS reagent program) and the mouse monoclonal antibodies against GFAP (Millipore) and Iba-1 (Wako). Double-immunocytochemical analysis was performed utilizing the Tyramide Signal Amplification™-Direct (Red) system (NEN Life Sciences, Boston, MA) to detect Iba-1 or GFAP and fluorescein isothiocyanate (FITC)-conjugated secondary antibodies (1:75, Vector) to detect Tat or gp120. Sections were imaged with a laser scanning confocal microscope BioRad Radiance 2000 (Hercules, CA) equipped with a Nikon E600FN Ellipse microscope (Japan) and using a Nikon Plan Apo 60x oil objective (NA 1.4; oil immersion). Statistical significance of differences in the expression of GFAP and Iba-1 between HIV-1 Tg rats and controls was determined by unpaired Student’s *t*-test.

### Gene expression profiling

Processing of Affymetrix microarrays was carried out as previously described, according to manufacturer’s procedures [125,126]. All analyses were performed with R statistical software. We analyzed 11 samples (5 HIV-1 Tg rats; 6 controls) hybridized on Affymetrix Rat 230.2. One additional HIV sample was removed as it showed lower quality than the other samples. Differential expression was computed with functions from the package limma and takes into account sample phenotype and batch. False Discovery Rates were computed by Benjamini & Hochberg method. Differential expression between HIV-1 and control rats identifies 614 and 4 probes differentially expressed at p-value < 0.01 and FDR < 0.05, respectively.

Pathways for GSEA analysis were from the MSigDb collection C2 that contains major pathway databases including KEGG, REACTOME and PID (http://www.broadinstitute.org/gsea/msigdb/index.jsp). We “humanized” the rat gene expression profiles by first selecting one probe per gene (the one with the highest coefficient of variation) and then kept the genes that had a unique human homologous gene (according to the database Homologene http://www.ncbi.nlm.nih.gov/homologene) for pathway analysis. Pathway differential activity was performed with GSEA using t-statistics obtained from the limma test as the reference list and pathway members as genesets; 24 pathways showed a p-value < 0.01. We report the pathways with the MSigDB terminology in the text and figures.

### RT-PCR

RT-PCR was carried out as previously described [125,127,128] with SYBR Green (BioRad) detection using an iQ5 Real-Time PCR Detection System (BioRad, Hercules, CA). The relative amounts of target mRNA were determined by the ΔCt method using β-actin for normalization [129]. Statistical significance of differences between HIV-1 Tg rats and controls was determined by unpaired Student’s *t*-test.

### Working memory

Rats were tested in the dark (<20 lux) during the nocturnal period using two identical T-Mazes constructed of black Plexiglas with no visual cues provided to minimize the influence of cataracts in HIV-1 Tg rats, as done by others [33]. Rats were first habituated to the experimenters over 4 days (5 min/day) as described in [29] and [30]. On test day, rats were allowed to alternate between the left and right goal arms of the T-maze in 15 trial sessions. Each session consisted of 1 forced trial followed by 14 free choice trials. Once the rat entered a particular goal arm (4 paws inside), a guillotine door was lowered to block entry to the opposite arm. The door was removed only after the rat returned to the start arm, thus allowing a new alternation trial to be started. If the rat did not enter one of the two arms after 120 seconds, the rat was placed in the previously chosen arm and the trial was considered as failed. If after 30 seconds the rat did not spontaneously return to the start box, the experimenter manually positioned the rat in the start box for the next trial. The spontaneous alternation behavior (SAB) and the response bias were calculated with the formula below where *n* = number of choices, *na’* = number of non-alternation responses, ρ_R_^2^ = probability of a right turn, ρ_L_^2^ = probability of a left turn [130]. The response bias corresponds to the tendency of an animal to favor one side over the other. The values of the response bias vary from 0.5 (no bias or same number of left and right choices) to 1.0 (full bias or exclusive left or right choices). Because animals with high response bias automatically exhibit low SAB that may be independent of any cognitive deficits, calculation of SAB without integrating the response bias may lead to wrong interpretations of the results by artificially producing low values below chance level. The formula was established so that a response bias of 0.5 (no bias) does not affect the calculation of SAB while a response of 1.0 (full bias) will neutralize the value of SAB to chance level (50%). This correction ensures that low levels of SAB are not due to a non-specific effect of response bias toward one side of the maze. The latency to leave the box and the latency to make a choice were also recorded.

SAB=501+n−na'ρL2+ρR2n;ResponseBia=ρL2+ρR2

## Abbreviations

cART: Combination antiretroviral therapy; CNS: Central nervous system; Col1A2: Procollagen type1-a2; COX-2: Cyclooxygenase-2; CSF: Cerebrospinal fluid; DAB: Diaminobenzidine; ERK1: Extracellular signal-regulated kinase 1; GFAP: Glial fibrillary acidic protein; GSEA: Gene Set Enrichment Analysis; HAD: HIV-associated dementia; HAND: HIV-associated neurocognitive disorders; HIVE: HIV encephalitis; IGF: Insulin-like growth factor; Igfbp: IGF binding protein; ILK: Integrin-linked kinase; ISG-15: Interferon stimulated gene 15; MAPK3: Mitogen-activated protein kinase 3; NRG1: Neuregulin-1; PGD2: Prostaglandin D2; PHLDA: Pleckstrin homology-like domain family A member 1; Ptgds: PGD2 synthase; SAB: Spontaneous alternation behavior; TDAG51: T cell death-associated gene 51; Tg: Transgenic.

## Competing interests

The authors declare that they have no competing interests.

## Authors’ contributions

PPS designed the study; VRC, CL, OG, MM, EM, carried out the studies; VRC, CL, OG, MM, GK, AC, EM, PPS wrote the paper. All authors read and approved the final manuscript.

## Supplementary Material

Additional file 1: Table S1 Top 614 differentially expressed probesets. Click here for file

Additional file 2: Table S2 Top 100 differentially expressed probesets. Click here for file

Additional file 3: Table S3 Top 23 differentially expressed pathways by GSEA. Click here for file

## References

[B1] Maschke M Kastrup O Esser S Ross B Hengge U Hufnagel A Incidence and prevalence of neurological disorders associated with HIV since the introduction of highly active antiretroviral therapy (HAART) J Neurol Neurosurg Psychiatry 2000 69 376 380 10945813 10.1136/jnnp.69.3.376 PMC1737101

[B2] D'Arminio Monforte A Cinque P Mocroft A Goebel FD Antunes F Katlama C Justesen US Vella S Kirk O Lundgren J Changing incidence of central nervous system diseases in the EuroSIDA cohort Ann Neurol 2004 55 320 328 14991809 10.1002/ana.10827

[B3] Everall IP Hansen LA Masliah E The shifting patterns of HIV encephalitis neuropathology Neurotox Res 2005 8 51 61 16260385 10.1007/BF03033819

[B4] Everall I Vaida F Khanlou N Lazzaretto D Achim C Letendre S Moore D Ellis R Cherne M Gelman B Morgello S Singer E Grant I Masliah E National NeuroAIDS Tissue Consortium (NNTC) Cliniconeuropathologic correlates of human immunodeficiency virus in the era of antiretroviral therapy J Neurovirol 2009 1 11 10.3109/13550280903131915 PMC3078805 20175693

[B5] Heaton RK Franklin DR Ellis RJ McCutchan JA Letendre SL Leblanc S Corkran SH Duarte NA Clifford DB Woods SP Collier AC Marra CM Morgello S Mindt MR Taylor MJ Marcotte TD Atkinson JH Wolfson T Gelman BB McArthur JC Simpson DM Abramson I Gamst A Fennema-Notestine C Jernigan TL Wong J Grant I CHARTER Group; HNRC Group HIV-associated neurocognitive disorders before and during the era of combination antiretroviral therapy: differences in rates, nature, and predictors J Neurovirol 2011 17 3 16 21174240 10.1007/s13365-010-0006-1 PMC3032197

[B6] Cysique LA Maruff P Brew BJ Prevalence and pattern of neuropsychological impairment in human immunodeficiency virus-infected/acquired immunodeficiency syndrome (HIV/AIDS) patients across pre- and post-highly active antiretroviral therapy eras: a combined study of two cohorts J Neurovirol 2004 10 350 357 15765806 10.1080/13550280490521078

[B7] Tozzi V Balestra P Serraino D Bellagamba R Corpolongo A Piselli P Lorenzini P Visco-Comandini U Vlassi C Quartuccio ME Giulianelli M Noto P Galgani S Ippolito G Antinori A Narciso P Neurocognitive impairment and survival in a cohort of HIV-infected patients treated with HAART AIDS Res Hum Retroviruses 2005 21 706 713 16131310 10.1089/aid.2005.21.706

[B8] Neuenburg JK Brodt HR Herndier BG Bickel M Bacchetti P Price RW Grant RM Schlote W HIV-related neuropathology, 1985 to 1999: rising prevalence of HIV encephalopathy in the era of highly active antiretroviral therapy J Acquir Immune Defic Syndr 2002 31 171 177 12394795 10.1097/00126334-200210010-00007

[B9] McArthur JC HIV dementia: an evolving disease J Neuroimmunol 2004 157 3 10 15579274 10.1016/j.jneuroim.2004.08.042

[B10] Brew BJ Evidence for a change in AIDS dementia complex in the era of highly active antiretroviral therapy and the possibility of new forms of AIDS dementia complex Aids 2004 18 Suppl 1 S75 S78 15075501

[B11] Gendelman HE Lipton SA Tardieu M Bukrinsky MI Nottet HS The neuropathogenesis of HIV-1 infection J Leukoc Biol 1994 56 389 398 8083614 10.1002/jlb.56.3.389

[B12] Gendelman HE Persidsky Y Ghorpade A Limoges J Stins M Fiala M Morrisett R The neuropathogenesis of the AIDS dementia complex AIDS 1997 11 Suppl A S35 S45 9451964

[B13] Patel CA Mukhtar M Harley S Kulkosky J Pomerantz RJ Lentiviral expression of HIV-1 Vpr induces apoptosis in human neurons J Neurovirol 2002 8 86 99 11935461 10.1080/13550280290049552

[B14] Jones GJ Barsby NL Cohen EA Holden J Harris K Dickie P Jhamandas J Power C HIV-1 Vpr causes neuronal apoptosis and in vivo neurodegeneration J Neurosci off J Soc Neurosci 2007 27 3703 3711 10.1523/JNEUROSCI.5522-06.2007 PMC6672409 17409234

[B15] van de Bovenkamp M Nottet HS Pereira CF Interactions of human immunodeficiency virus-1 proteins with neurons: possible role in the development of human immunodeficiency virus-1-associated dementia Eur J Clin Invest 2002 32 619 627 12190962 10.1046/j.1365-2362.2002.01029.x

[B16] Adamson DC Kopnisky KL Dawson TM Dawson VL Mechanisms and structural determinants of HIV-1 coat protein, gp41-induced neurotoxicity J Neurosci off J Soc Neurosci 1999 19 64 71 10.1523/JNEUROSCI.19-01-00064.1999 PMC6782354 9870939

[B17] Ellis R Langford D Masliah E HIV and antiretroviral therapy in the brain: neuronal injury and repair Nat Rev Neurosci 2007 8 33 44 17180161 10.1038/nrn2040

[B18] Masliah E Heaton RK Marcotte TD Ellis RJ Wiley CA Mallory M Achim CL McCutchan JA Nelson JA Atkinson JH Grant I Dendritic injury is a pathological substrate for human immunodeficiency virus-related cognitive disorders. HNRC Group. The HIV Neurobehavioral Research Center Ann Neurol 1997 42 963 972 9403489 10.1002/ana.410420618

[B19] Iskander S Walsh KA Hammond RR Human CNS cultures exposed to HIV-1 gp120 reproduce dendritic injuries of HIV-1-associated dementia J Neuroinflammation 2004 1 7 15285795 10.1186/1742-2094-1-7 PMC483060

[B20] Toggas SM Masliah E Rockenstein EM Rall GF Abraham CR Mucke L Central nervous system damage produced by expression of the HIV-1 coat protein gp120 in transgenic mice Nature 1994 367 188 193 8114918 10.1038/367188a0

[B21] Kim BO Liu Y Ruan Y Xu ZC Schantz L He JJ Neuropathologies in transgenic mice expressing human immunodeficiency virus type 1 Tat protein under the regulation of the astrocyte-specific glial fibrillary acidic protein promoter and doxycycline Am J Pathol 2003 162 1693 1707 12707054 10.1016/S0002-9440(10)64304-0 PMC1851199

[B22] Reid W Sadowska M Denaro F Rao S Foulke J Jr Hayes N Jones O Doodnauth D Davis H Sill A O'Driscoll P Huso D Fouts T Lewis G Hill M Kamin-Lewis R Wei C Ray P Gallo RC Reitz M Bryant J An HIV-1 transgenic rat that develops HIV-related pathology and immunologic dysfunction Proc Natl Acad Sci U S A 2001 98 9271 9276 11481487 10.1073/pnas.161290298 PMC55410

[B23] Royal W 3rd Zhang L Guo M Jones O Davis H Bryant JL Immune activation, viral gene product expression and neurotoxicity in the HIV-1 transgenic rat J Neuroimmunol 2012 247 16 24 22503372 10.1016/j.jneuroim.2012.03.015 PMC3351529

[B24] Hoang V Withers-Ward E Camerini D Nonprimate models of HIV-1 infection and pathogenesis Adv Pharmacol 2008 56 399 422 18086419 10.1016/S1054-3589(07)56013-8

[B25] Dickie P Felser J Eckhaus M Bryant J Silver J Marinos N Notkins AL HIV-associated nephropathy in transgenic mice expressing HIV-1 genes Virology 1991 185 109 119 1926769 10.1016/0042-6822(91)90759-5

[B26] Wei P Garber ME Fang SM Fischer WH Jones KA A novel CDK9-associated C-type cyclin interacts directly with HIV-1 Tat and mediates its high-affinity, loop-specific binding to TAR RNA Cell 1998 92 451 462 9491887 10.1016/s0092-8674(00)80939-3

[B27] Reid W Abdelwahab S Sadowska M Huso D Neal A Ahearn A Bryant J Gallo RC Lewis GK Reitz M HIV-1 transgenic rats develop T cell abnormalities Virology 2004 321 111 119 15033570 10.1016/j.virol.2003.12.010

[B28] Subramanian A Tamayo P Mootha VK Mukherjee S Ebert BL Gillette MA Paulovich A Pomeroy SL Golub TR Lander ES Mesirov JP Gene set enrichment analysis: a knowledge-based approach for interpreting genome-wide expression profiles Proc Natl Acad Sci USA 2005 102 15545 15550 16199517 10.1073/pnas.0506580102 PMC1239896

[B29] Gerlai R A new continuous alternation task in T-maze detects hippocampal dysfunction in mice. A strain comparison and lesion study Behav Brain Res 1998 95 91 101 9754881 10.1016/s0166-4328(97)00214-3

[B30] Gerlai R Shinsky N Shih A Williams P Winer J Armanini M Cairns B Winslow J Gao W Phillips HS Regulation of learning by EphA receptors: a protein targeting study J Neurosci off j Soc Neurosci 1999 19 9538 9549 10.1523/JNEUROSCI.19-21-09538.1999 PMC6782889 10531456

[B31] Clark RE Zola SM Squire LR Impaired recognition memory in rats after damage to the hippocampus J Neurosci Off J Soci Neurosci 2000 20 8853 8860 10.1523/JNEUROSCI.20-23-08853.2000 PMC6773055 11102494

[B32] Johnson CT Olton DS Gage FH 3rd Jenko PG Damage to hippocampus and hippocampal connections: effects on DRL and spontaneous alternation J Comp Physiol Psychol 1977 91 508 522 874119 10.1037/h0077346

[B33] Futter JE Aggleton JP How rats perform spatial working memory tasks: limitations in the use of egocentric and idiothetic working memory Q J Exp Psychol (Colchester) 2006 59 77 99 10.1080/02724990544000068 16556560

[B34] Stone WS Rudd RJ Parsons MW Gold PE Memory scores in middle-aged rats predict later deficits in memory, paradoxical sleep, and blood glucose regulation in old age Exp Aging Res 1997 23 287 300 9248821 10.1080/03610739708254285

[B35] Buhot MC Dubayle D Malleret G Javerzat S Segu L Exploration, anxiety, and spatial memory in transgenic anophthalmic mice Behav Neurosci 2001 115 455 467 11345970

[B36] Lashomb AL Vigorito M Chang SL Further characterization of the spatial learning deficit in the human immunodeficiency virus-1 transgenic rat J Neurovirol 2009 15 14 24 19085205 10.1080/13550280802232996

[B37] Vigorito M LaShomb AL Chang SL Spatial learning and memory in HIV-1 transgenic rats J Neuroimmune Pharmacol 2007 2 319 328 18040850 10.1007/s11481-007-9078-y

[B38] Fields J Dumaop W Adame A Ellis RJ Letendre S Grant I Masliah E Alterations in the Levels of Vesicular Trafficking Proteins Involved in HIV Replication in the Brains and CSF of Patients with HIV-associated Neurocognitive Disorders J Neuroimmune Pharmacol 2013 8 5 1197 209 doi:10.1007/s11481-013-9511-3. 24292993 10.1007/s11481-013-9511-3 PMC3973444

[B39] Wang RG Kaul M Zhang DX Interferon-stimulated gene 15 as a general marker for acute and chronic neuronal injuries Sheng Li Xue Bao 2012 64 577 583 23090498 PMC3587786

[B40] Katsounas A Hubbard JJ Wang CH Zhang X Dou D Shivakumar B Winter S Schlaak JF Lempicki RA Masur H Polis M Kottilil S Osinusi A High interferon-stimulated gene ISG-15 expression affects HCV treatment outcome in patients co-infected with HIV and HCV J Med Virol 2013 85 959 963 23588721 10.1002/jmv.23576

[B41] Meeker RB Poulton W Markovic-Plese S Hall C Robertson K Protein changes in CSF of HIV-infected patients: evidence for loss of neuroprotection J Neurovirol 2011 17 258 273 21556959 10.1007/s13365-011-0034-5 PMC3166824

[B42] Froldi M Castagna A Parma M Piona A Tedeschi A Miadonna A Lorini M Orazio EN Lazzarin A Mediator release in cerebrospinal fluid of human immunodeficiency virus-positive patients with central nervous system involvement J Neuroimmunol 1992 38 155 161 1315794 10.1016/0165-5728(92)90100-y

[B43] Masliah E Roberts ES Langford D Everall I Crews L Adame A Rockenstein E Fox HS Patterns of gene dysregulation in the frontal cortex of patients with HIV encephalitis J Neuroimmunol 2004 157 163 175 15579294 10.1016/j.jneuroim.2004.08.026

[B44] Shaw KN Commins S O'Mara SM Cyclooxygenase inhibition attenuates endotoxin-induced spatial learning deficits, but not an endotoxin-induced blockade of long-term potentiation Brain Res 2005 1038 231 237 15757639 10.1016/j.brainres.2005.01.035

[B45] Basselin M Ramadan E Igarashi M Chang L Chen M Kraft AD Harry GJ Rapoport SI Imaging upregulated brain arachidonic acid metabolism in HIV-1 transgenic rats J Cereb Blood Flow Metab 2011 31 486 493 20664612 10.1038/jcbfm.2010.111 PMC2992106

[B46] Mohri I Taniike M Taniguchi H Kanekiyo T Aritake K Inui T Fukumoto N Eguchi N Kushi A Sasai H Kanaoka Y Ozono K Narumiya S Suzuki K Urade Y Prostaglandin D2-mediated microglia/astrocyte interaction enhances astrogliosis and demyelination in twitcher J Neurosci Off J Soc Neurosci 2006 26 4383 4393 10.1523/JNEUROSCI.4531-05.2006 PMC6673986 16624958

[B47] Kalaria RN Pax AB Increased collagen content of cerebral microvessels in Alzheimer's disease Brain Res 1995 705 349 352 8821769 10.1016/0006-8993(95)01250-8

[B48] van Horssen J Bo L Dijkstra CD de Vries HE Extensive extracellular matrix depositions in active multiple sclerosis lesions Neurobiol Dis 2006 24 484 491 17005408 10.1016/j.nbd.2006.08.005

[B49] Veznedaroglu E Van Bockstaele EJ O'Connor MJ Extravascular collagen in the human epileptic brain: a potential substrate for aberrant cell migration in cases of temporal lobe epilepsy J Neurosurg 2002 97 1125 1130 12450035 10.3171/jns.2002.97.5.1125

[B50] Gelman BB Chen T Lisinicchia JG Soukup VM Carmical JR Starkey JM Masliah E Commins DL Brandt D Grant I Singer EJ Levine AJ Miller J Winkler JM Fox HS Luxon BA Morgello S National NeuroAIDS Tissue Consortium The National NeuroAIDS Tissue Consortium brain gene array: two types of HIV-associated neurocognitive impairment PLoS One 2012 7 e46178 23049970 10.1371/journal.pone.0046178 PMC3458860

[B51] Pocernich CB Poon HF Boyd-Kimball D Lynn BC Nath A Klein JB Butterfield DA Proteomic analysis of oxidatively modified proteins induced by the mitochondrial toxin 3-nitropropionic acid in human astrocytes expressing the HIV protein tat Brain Res Mol Brain Res 2005 133 299 306 15710247 10.1016/j.molbrainres.2004.10.024

[B52] Landi S Putignano E Boggio EM Giustetto M Pizzorusso T Ratto GM The short-time structural plasticity of dendritic spines is altered in a model of Rett syndrome Scientific Reports 2011 1 45 22355564 10.1038/srep00045 PMC3216532

[B53] Barros CS Calabrese B Chamero P Roberts AJ Korzus E Lloyd K Stowers L Mayford M Halpain S Muller U Impaired maturation of dendritic spines without disorganization of cortical cell layers in mice lacking NRG1/ErbB signaling in the central nervous system Proc Natl Acad Sci USA 2009 106 4507 4512 19240213 10.1073/pnas.0900355106 PMC2657442

[B54] Grant A Hoops D Labelle-Dumais C Prevost M Rajabi H Kolb B Stewart J Arvanitogiannis A Flores C Netrin-1 receptor-deficient mice show enhanced mesocortical dopamine transmission and blunted behavioural responses to amphetamine Eur J Neurosci 2007 26 3215 3228 18005074 10.1111/j.1460-9568.2007.05888.x

[B55] Benarroch EE Insulin-like growth factors in the brain and their potential clinical implications Neurology 2012 79 2148 2153 23170013 10.1212/WNL.0b013e3182752eef

[B56] Szczesny E Slusarczyk J Glombik K Budziszewska B Kubera M Lason W Basta-Kaim A Possible contribution of IGF-1 to depressive disorder Pharmacol Reports PR 2013 65 1622 1631 10.1016/s1734-1140(13)71523-8 24553010

[B57] Von Bohlen Und Halbach O Unsicker K Neurotrophic support of midbrain dopaminergic neurons Adv Exp Med Biol 2009 651 73 80 19731552 10.1007/978-1-4419-0322-8_7

[B58] Clemmons DR Busby WH Arai T Nam TJ Clarke JB Jones JI Ankrapp DK Role of insulin-like growth factor binding proteins in the control of IGF actions Progress Growth Factor Res 1995 6 357 366 10.1016/0955-2235(95)00013-5 8817679

[B59] Clemmons DR Jones JI Busby WH Wright G Role of insulin-like growth factor binding proteins in modifying IGF actions Ann N Y Acad Sci 1993 692 10 21 7692784 10.1111/j.1749-6632.1993.tb26201.x

[B60] Besset V Le Magueresse-Battistoni B Collette J Benahmed M Tumor necrosis factor alpha stimulates insulin-like growth factor binding protein 3 expression in cultured porcine Sertoli cells Endocrinology 1996 137 296 303 8536626 10.1210/endo.137.1.8536626

[B61] Scharla SH Strong DD Mohan S Chevalley T Linkhart TA Effect of tumor necrosis factor-alpha on the expression of insulin-like growth factor I and insulin-like growth factor binding protein 4 in mouse osteoblasts Eur J Endocrinol/Eur Federation Endocrine Societies 1994 131 293 301 10.1530/eje.0.1310293 7522842

[B62] Street ME Ziveri MA Spaggiari C Viani I Volta C Grzincich GL Virdis R Bernasconi S Inflammation is a modulator of the insulin-like growth factor (IGF)/IGF-binding protein system inducing reduced bioactivity of IGFs in cystic fibrosis Eur J Endocrinol / Eur Federation Endocrine Societies 2006 154 47 52 10.1530/eje.1.02064 16381990

[B63] Tazearslan C Huang J Barzilai N Suh Y Impaired IGF1R signaling in cells expressing longevity-associated human IGF1R alleles Aging cell 2011 10 551 554 21388493 10.1111/j.1474-9726.2011.00697.x PMC3094477

[B64] Xi ZQ Wang LY Sun JJ Liu XZ Zhu X Xiao F Guan LF Li JM Wang L Wang XF TDAG51 in the anterior temporal neocortex of patients with intractable epilepsy Neurosci Lett 2007 425 53 58 17870236 10.1016/j.neulet.2007.08.016

[B65] Basseri S Lhotak S Fullerton MD Palanivel R Jiang H Lynn EG Ford RJ Maclean KN Steinberg GR Austin RC Loss of TDAG51 results in mature-onset obesity, hepatic steatosis, and insulin resistance by regulating lipogenesis Diabetes 2013 62 158 169 22961087 10.2337/db12-0256 PMC3526025

[B66] Murata T Sato T Kamoda T Moriyama H Kumazawa Y Hanada N Differential susceptibility to hydrogen sulfide-induced apoptosis between PHLDA1-overexpressing oral cancer cell lines and oral keratinocytes: Role of PHLDA1 as an apoptosis suppressor Exp Cell Res 2014 320 247 257 24270013 10.1016/j.yexcr.2013.10.023

[B67] Park ES Kim J Ha TU Choi JS Soo Hong K Rho J TDAG51 deficiency promotes oxidative stress-induced apoptosis through the generation of reactive oxygen species in mouse embryonic fibroblasts Exp Mol Med 2013 45 e35 23928855 10.1038/emm.2013.67 PMC3789259

[B68] Park CG Lee SY Kandala G Lee SY Choi Y A novel gene product that couples TCR signaling to Fas(CD95) expression in activation-induced cell death Immunity 1996 4 583 591 8673705 10.1016/s1074-7613(00)80484-7

[B69] Mei L Xiong WC Neuregulin 1 in neural development, synaptic plasticity and schizophrenia Nat Rev Neurosci 2008 9 437 452 18478032 10.1038/nrn2392 PMC2682371

[B70] Lai Wing Sun K Correia JP Kennedy TE Netrins: versatile extracellular cues with diverse functions Development 2011 138 2153 2169 21558366 10.1242/dev.044529

[B71] Horn KE Glasgow SD Gobert D Bull SJ Luk T Girgis J Tremblay ME McEachern D Bouchard JF Haber M Hamel E Krimpenfort P Murai KK Berns A Doucet G Chapman CA Ruthazer ES Kennedy TE DCC expression by neurons regulates synaptic plasticity in the adult brain Cell Rep 2013 3 173 185 23291093 10.1016/j.celrep.2012.12.005

[B72] Llambi F Lourenco FC Gozuacik D Guix C Pays L Del Rio G Kimchi A Mehlen P The dependence receptor UNC5H2 mediates apoptosis through DAP-kinase EMBO J 2005 24 1192 1201 15729359 10.1038/sj.emboj.7600584 PMC556396

[B73] Barallobre MJ Pascual M Del Rio JA Soriano E The Netrin family of guidance factors: emphasis on Netrin-1 signalling Brain Res Brain Res Rev 2005 49 22 47 15960985 10.1016/j.brainresrev.2004.11.003

[B74] Bradford D Faull RL Curtis MA Cooper HM Characterization of the netrin/RGMa receptor neogenin in neurogenic regions of the mouse and human adult forebrain J Comp Neurol 2010 518 3237 3253 20575069 10.1002/cne.22397

[B75] Staquicini FI Dias-Neto E Li J Snyder EY Sidman RL Pasqualini R Arap W Discovery of a functional protein complex of netrin-4, laminin gamma1 chain, and integrin alpha6beta1 in mouse neural stem cells Proc Natl Acad Sci U S A 2009 106 2903 2908 19193855 10.1073/pnas.0813286106 PMC2635839

[B76] Cayre M Courtes S Martineau F Giordano M Arnaud K Zamaron A Durbec P Netrin 1 contributes to vascular remodeling in the subventricular zone and promotes progenitor emigration after demyelination Development 2013 140 3107 3117 23824572 10.1242/dev.092999

[B77] Peng H Sun L Jia B Lan X Zhu B Wu Y Zheng J HIV-1-infected and immune-activated macrophages induce astrocytic differentiation of human cortical neural progenitor cells via the STAT3 pathway PLoS One 2011 6 e19439 21637744 10.1371/journal.pone.0019439 PMC3103496

[B78] Swiech L Perycz M Malik A Jaworski J Role of mTOR in physiology and pathology of the nervous system Biochim Biophys Acta 2008 1784 116 132 17913600 10.1016/j.bbapap.2007.08.015

[B79] Germain M Nguyen AP Khacho M Patten DA Screaton RA Park DS Slack RS LKB1-regulated adaptive mechanisms are essential for neuronal survival following mitochondrial dysfunction Hum Mol Genet 2013 22 952 962 23187960 10.1093/hmg/dds500

[B80] Cross DA Alessi DR Cohen P Andjelkovich M Hemmings BA Inhibition of glycogen synthase kinase-3 by insulin mediated by protein kinase B Nature 1995 378 785 789 8524413 10.1038/378785a0

[B81] Mocchetti I Bachis A Campbell LA Avdoshina V Implementing Neuronal Plasticity in NeuroAIDS: the Experience of Brain-derived Neurotrophic Factor and other Neurotrophic Factors J Neuroimmune Pharmacol Off J Soc NeuroImmune Pharmacol 2013 9 2 80 91 doi:10.1007/s11481-013-9488-y 10.1007/s11481-013-9488-y PMC3844100 23832285

[B82] Woodbury ME Ikezu T Fibroblast Growth Factor-2 Signaling in Neurogenesis and Neurodegeneration J Neuroimmune Pharmacol Off J Soc NeuroImmune Pharmacol 2013 9 2 92 101 doi:10.1007/s11481-013-9501-5 10.1007/s11481-013-9501-5 PMC4109802 24057103

[B83] Fields J Dumaop W Langford TD Rockenstein E Masliah E Role of Neurotrophic Factor Alterations in the Neurodegenerative Process in HIV Associated Neurocognitive Disorders J Neuroimmune Pharmacol Off J Soc NeuroImmune Pharmacol 2014 9 2 102 116 doi:10.1007/s11481-013-9520-2. 10.1007/s11481-013-9520-2 PMC3973421 24510686

[B84] Sanna PP Cammalleri M Berton F Simpson C Lutjens R Bloom FE Francesconi W Phosphatidylinositol 3-kinase is required for the expression but not for the induction or the maintenance of long-term potentiation in the hippocampal CA1 region J Neurosci Off J Soc for Neurosci 2002 22 3359 3365 10.1523/JNEUROSCI.22-09-03359.2002 PMC6758361 11978812

[B85] Cammalleri M Lutjens R Berton F King AR Simpson C Francesconi W Sanna PP Time-restricted role for dendritic activation of the mTOR-p70S6K pathway in the induction of late-phase long-term potentiation in the CA1 Proc Natl Acad Sci USA 2003 100 14368 14373 14623952 10.1073/pnas.2336098100 PMC283598

[B86] Tang SJ Reis G Kang H Gingras AC Sonenberg N Schuman EM A rapamycin-sensitive signaling pathway contributes to long-term synaptic plasticity in the hippocampus Proc Natl Acad Sci U S A 2002 99 467 472 11756682 10.1073/pnas.012605299 PMC117583

[B87] Diaz-Ruiz O Navarro L Mendez-Diaz M Galicia O Elder JH Sanna PP Drucker-Colin R Prospero-Garcia O Inhibition of the ERK pathway prevents HIVgp120-induced REM sleep increase Brain Res 2001 913 78 81 11532249 10.1016/s0006-8993(01)02745-7

[B88] Van Veldhoven PP Biochemistry and genetics of inherited disorders of peroxisomal fatty acid metabolism J Lipid Res 2010 51 2863 2895 20558530 10.1194/jlr.R005959 PMC2936746

[B89] Ivashchenko O Van Veldhoven PP Brees C Ho YS Terlecky SR Fransen M Intraperoxisomal redox balance in mammalian cells: oxidative stress and interorganellar cross-talk Mol Biol Cell 2011 22 1440 1451 21372177 10.1091/mbc.E10-11-0919 PMC3084667

[B90] Louboutin JP Agrawal L Reyes BA Van Bockstaele EJ Strayer DS HIV-1 gp120-induced injury to the blood–brain barrier: role of metalloproteinases 2 and 9 and relationship to oxidative stress J Neuropathol Exp Neurol 2010 69 801 816 20613638 10.1097/NEN.0b013e3181e8c96f PMC4707960

[B91] Masliah E Ge N Mucke L Pathogenesis of HIV-1 associated neurodegeneration Crit Rev Neurobiol 1996 10 57 67 8853954 10.1615/critrevneurobiol.v10.i1.30

[B92] Cutler RG Haughey NJ Tammara A McArthur JC Nath A Reid R Vargas DL Pardo CA Mattson MP Dysregulation of sphingolipid and sterol metabolism by ApoE4 in HIV dementia Neurology 2004 63 626 630 15326233 10.1212/01.wnl.0000134662.19883.06

[B93] Deicken RF Hubesch B Jensen PC Sappey-Marinier D Krell P Wisniewski A Vanderburg D Parks R Fein G Weiner MW Alterations in brain phosphate metabolite concentrations in patients with human immunodeficiency virus infection Arch Neurol 1991 48 203 209 1993012 10.1001/archneur.1991.00530140099022

[B94] Uzasci L Nath A Cotter R Oxidative stress and the HIV-infected brain proteome J Neuroimmune Pharmacol Off J Soc NeuroImmune Pharmacol 2013 8 1167 1180 10.1007/s11481-013-9444-x PMC3714334 23475542

[B95] Margottin F Bour SP Durand H Selig L Benichou S Richard V Thomas D Strebel K Benarous R A novel human WD protein, h-beta TrCp, that interacts with HIV-1 Vpu connects CD4 to the ER degradation pathway through an F-box motif Mol Cell 1998 1 565 574 9660940 10.1016/s1097-2765(00)80056-8

[B96] Li YP Protein B23 is an important human factor for the nucleolar localization of the human immunodeficiency virus protein Tat J Virol 1997 71 4098 4102 9094689 10.1128/jvi.71.5.4098-4102.1997 PMC191564

[B97] Bisikirska BC Adam SJ Alvarez MJ Rajbhandari P Cox R Lefebvre C Wang K Rieckhof GE Felsher DW Califano A STK38 is a critical upstream regulator of MYC's oncogenic activity in human B-cell lymphoma Oncogene 2013 32 5283 5291 23178486 10.1038/onc.2012.543 PMC3715597

[B98] Conti B Maier R Barr AM Morale MC Lu X Sanna PP Bilbe G Hoyer D Bartfai T Region-specific transcriptional changes following the three antidepressant treatments electro convulsive therapy, sleep deprivation and fluoxetine Mol Psychiatry 2006 12 2 167 189 17033635 10.1038/sj.mp.4001897

[B99] Huang YC The role of in vitro gene expression profiling in particulate matter health research J Toxicol Environ Health B Crit Rev 2013 16 381 394 24151968 10.1080/10937404.2013.832649

[B100] Letendre S Central nervous system complications in HIV disease: HIV-associated neurocognitive disorder Top Antivir Med 2011 19 137 142 22156215 PMC4666587

[B101] Nabha L Duong L Timpone J HIV-associated neurocognitive disorders: perspective on management strategies Drugs 2013 73 893 905 23733447 10.1007/s40265-013-0059-6 PMC3735343

[B102] Schifitto G Navia BA Yiannoutsos CT Marra CM Chang L Ernst T Jarvik JG Miller EN Singer EJ Ellis RJ Kolson DL Simpson D Nath A Berger J Shriver SL Millar LL Colquhoun D Lenkinski R Gonzalez RG Lipton SA Adult AIDS Clinical Trial Group (ACTG) 301; 700 Teams; HIV MRS Consortium Memantine and HIV-associated cognitive impairment: a neuropsychological and proton magnetic resonance spectroscopy study AIDS 2007 21 1877 1886 17721095 10.1097/QAD.0b013e32813384e8

[B103] Zhao Y Navia BA Marra CM Singer EJ Chang L Berger J Ellis RJ Kolson DL Simpson D Miller EN Lipton SA Evans SR Schifitto G Adult Aids Clinical Trial Group (ACTG) 301 Team Memantine for AIDS dementia complex: open-label report of ACTG 301 HIV Clin Trials 2010 11 59 67 20400412 10.1310/hct1101-59 PMC2981797

[B104] Fabbrini G Abbruzzese G Marconi S Zappia M Selegiline: a reappraisal of its role in Parkinson disease Clin Neuropharmacol 2012 35 134 140 22592509 10.1097/WNF.0b013e318255838b

[B105] Shulman KI Herrmann N Walker SE Current place of monoamine oxidase inhibitors in the treatment of depression CNS drugs 2013 27 789 797 23934742 10.1007/s40263-013-0097-3

[B106] Breitbart W Rosenfeld B Kaim M Funesti-Esch J A randomized, double-blind, placebo-controlled trial of psychostimulants for the treatment of fatigue in ambulatory patients with human immunodeficiency virus disease Arch Intern Med 2001 161 411 420 11176767 10.1001/archinte.161.3.411

[B107] Fiala M Liu QN Sayre J Pop V Brahmandam V Graves MC Vinters HV Cyclooxygenase-2-positive macrophages infiltrate the Alzheimer's disease brain and damage the blood–brain barrier Eur J Clin Investig 2002 32 360 371 12027877 10.1046/j.1365-2362.2002.00994.x

[B108] Graves MC Fiala M Dinglasan LA Liu NQ Sayre J Chiappelli F van Kooten C Vinters HV nflammation in amyotrophic lateral sclerosis spinal cord and brain is mediated by activated macrophages, mast cells and T cells Amyotrop Lateral Sclerosis Motor Neuron Disorders Off Publ World Fed Neurol Res Group Motor Neuron Dis 2004 5 213 219 10.1080/14660820410020286 15799549

[B109] Pettersen FO Torheim EA Dahm AE Aaberge IS Lind A Holm M Aandahl EM Sandset PM Tasken K Kvale D An exploratory trial of cyclooxygenase type 2 inhibitor in HIV-1 infection: downregulated immune activation and improved T cell-dependent vaccine responses J Virol 2011 85 6557 6566 21490090 10.1128/JVI.00073-11 PMC3126508

[B110] Ebert AD Beres AJ Barber AE Svendsen CN Human neural progenitor cells over-expressing IGF-1 protect dopamine neurons and restore function in a rat model of Parkinson's disease Exp Neurol 2008 209 213 223 18061591 10.1016/j.expneurol.2007.09.022

[B111] Quesada A Lee BY Micevych PE PI3 kinase/Akt activation mediates estrogen and IGF-1 nigral DA neuronal neuroprotection against a unilateral rat model of Parkinson's disease Dev Neurobiol 2008 68 632 644 18278798 10.1002/dneu.20609 PMC2667142

[B112] Carro E Trejo JL Gerber A Loetscher H Torrado J Metzger F Torres-Aleman I Therapeutic actions of insulin-like growth factor I on APP/PS2 mice with severe brain amyloidosis Neurobiol Aging 2006 27 1250 1257 16183170 10.1016/j.neurobiolaging.2005.06.015

[B113] Carro E Trejo JL Gomez-Isla T LeRoith D Torres-Aleman I Serum insulin-like growth factor I regulates brain amyloid-beta levels Nat Med 2002 8 1390 1397 12415260 10.1038/nm1202-793

[B114] Bassil F Fernagut PO Bezard E Meissner WG Insulin, IGF-1 and GLP-1 signaling in neurodegenerative disorders: Targets for disease modification? Prog Neurobiol 2014 118C 1 18 doi:10.1016/j.pneurobio.2014.02.005 24582776 10.1016/j.pneurobio.2014.02.005

[B115] Kang YJ Digicaylioglu M Russo R Kaul M Achim CL Fletcher L Masliah E Lipton SA Erythropoietin plus insulin-like growth factor-I protects against neuronal damage in a murine model of human immunodeficiency virus-associated neurocognitive disorders Ann Neurol 2010 68 342 352 20818790 10.1002/ana.22070 PMC3733362

[B116] Cabou C Campistron G Marsollier N Leloup C Cruciani-Guglielmacci C Penicaud L Drucker DJ Magnan C Burcelin R Brain glucagon-like peptide-1 regulates arterial blood flow, heart rate, and insulin sensitivity Diabetes 2008 57 2577 2587 18633100 10.2337/db08-0121 PMC2551665

[B117] Li Y Duffy KB Ottinger MA Ray B Bailey JA Holloway HW Tweedie D Perry T Mattson MP Kapogiannis D Sambamurti K Lahiri DK Greig NH GLP-1 receptor stimulation reduces amyloid-beta peptide accumulation and cytotoxicity in cellular and animal models of Alzheimer's disease J Alzheimer's Dis JAD 2010 19 1205 1219 10.3233/JAD-2010-1314 PMC2948479 20308787

[B118] Bertilsson G Patrone C Zachrisson O Andersson A Dannaeus K Heidrich J Kortesmaa J Mercer A Nielsen E Ronnholm H Wikstrom L Peptide hormone exendin-4 stimulates subventricular zone neurogenesis in the adult rodent brain and induces recovery in an animal model of Parkinson's disease J Neurosci Res 2008 86 326 338 17803225 10.1002/jnr.21483

[B119] Harkavyi A Abuirmeileh A Lever R Kingsbury AE Biggs CS Whitton PS Glucagon-like peptide 1 receptor stimulation reverses key deficits in distinct rodent models of Parkinson's disease J Neuroinflammation 2008 5 19 18492290 10.1186/1742-2094-5-19 PMC2426681

[B120] Gespach C Guidance for life, cell death, and colorectal neoplasia by netrin dependence receptors Adv Cancer Res 2012 114 87 186 22588057 10.1016/B978-0-12-386503-8.00004-1

[B121] Deng C Pan B Engel M Huang XF Neuregulin-1 signalling and antipsychotic treatment: potential therapeutic targets in a schizophrenia candidate signalling pathway Psychopharmacol 2013 226 201 215 10.1007/s00213-013-3003-2 23389757

[B122] Contreras X Barboric M Lenasi T Peterlin BM HMBA releases P-TEFb from HEXIM1 and 7SK snRNA via PI3K/Akt and activates HIV transcription PLoS Pathog 2007 3 1459 1469 17937499 10.1371/journal.ppat.0030146 PMC2014796

[B123] Doyon G Sobolewski MD Huber K McMahon D Mellors JW Sluis-Cremer N Discovery of a small molecule agonist of phosphatidylinositol 3-kinase p110alpha that reactivates latent HIV-1 PLoS One 2014 9 e84964 24489654 10.1371/journal.pone.0084964 PMC3906007

[B124] Patrick C Crews L Desplats P Dumaop W Rockenstein E Achim CL Everall IP Masliah E Increased CDK5 expression in HIV encephalitis contributes to neurodegeneration via tau phosphorylation and is reversed with Roscovitine Am J Pathol 2011 178 1646 1661 21435449 10.1016/j.ajpath.2010.12.033 PMC3078446

[B125] Ahmed SH Lutjens R van der Stap LD Lekic D Romano-Spica V Morales M Koob GF Repunte-Canonigo V Sanna PP Gene expression evidence for remodeling of lateral hypothalamic circuitry in cocaine addiction Proc Natl Acad Sci USA 2005 102 11533 11538 16076954 10.1073/pnas.0504438102 PMC1183574

[B126] Repunte-Canonigo V van der Stap LD Chen J Sabino V Wagner U Zorrilla EP Schumann G Roberts AJ Sanna PP Genome-wide gene expression analysis identifies K-ras as a regulator of alcohol intake Brain Res 2010 1339 1 10 20388501 10.1016/j.brainres.2010.03.063 PMC2925131

[B127] Repunte-Canonigo V Lutjens R van der Stap LD Sanna PP Increased expression of protein kinase A inhibitor alpha (PKI-alpha) and decreased PKA-regulated genes in chronic intermittent alcohol exposure Brain Res 2007 1138 48 56 17270154 10.1016/j.brainres.2006.09.115 PMC4485929

[B128] Repunte-Canonigo V Chen J Lefebvre C Kawamura T Kreifeldt M Basson O Roberts AJ Sanna PP MeCP2 regulates ethanol sensitivity and intake Addict Biol 2013 doi:10.1111/adb.12047 10.1111/adb.12047 PMC3692583 23448145

[B129] Livak KJ Schmittgen TD Analysis of relative gene expression data using real-time quantitative PCR and the 2(−Delta Delta C(T)) Method Methods 2001 25 402 408 11846609 10.1006/meth.2001.1262

[B130] Douglas RJ Raphelson AC Spontaneous alternation and septal lesions J Comp Physiol Psychol 1966 62 320 322 5338926 10.1037/h0023657

