# Supplementary material for: Gene expression changes consistent with neuroAIDS and impaired working memory in HIV-1 transgenic rats
Source: Mol Neurodegener. 2014 Jul 1;9:26. doi: 10.1186/1750-1326-9-26 (PMC4107468; doi:10.1186/1750-1326-9-26)
Supplement: Additional file 3: Table S3 — Top 23 differentially expressed pathways by GSEA. [file 1750-1326-9-26-S3.docx]

| MSigDB Pathway | ES | NES | p-value | genes in leading edge |
| --- | --- | --- | --- | --- |
| KEGG_HUNTINGTONS_DISEASE | -0.31 | -3.93 | 1.00E-03 | AP2B1 ATP5A1 ATP5D ATP5G3 BDNF CASP8 CASP9 COX1 COX2 COX5B COX6A1 COX7C COX8A CREB3L4 CREB5 CYTB DNAL4 EP300 GNAQ GRIN2B GRM5 HIP1 ITPR1 NDUFA4 NDUFA5 NDUFAB1 NDUFB7 NDUFC1 NDUFS3 NDUFV1 NDUFV3 NRF1 PLCB2 POLR2A POLR2E POLR2F POLR2J POLR2L PPARG REST SDHC SDHD SIN3A SLC25A31 TP53 UQCR11 UQCRC1 UQCRQ |
| PID_MTOR_4PATHWAY | -0.46 | -3.76 | 1.00E-03 | AKT1 AKT1S1 CCNE1 CDK2 DDIT4 EIF4B MAP2K2 MAPK1 MAPK3 MLST8 MTOR PLD1 PRR5 PXN RAC1 RAF1 RPS6KA1 RPTOR SFN ULK1 YWHAH YWHAZ YY1 |
| REACTOME_INTERFERON_ALPHA_BETA_SIGNALING | 0.49 | 3.35 | 1.00E-03 | IFI27 IFI35 IFIT2 IFITM1 IRF6 IRF7 IRF9 ISG15 MX1 OAS1 PSMB8 SOCS3 USP18 |
| REACTOME_REGULATION_OF_INSULIN_LIKE_GROWTH_FACTOR_IGF_ACTIVITY_BY_INSULIN_LIKE_GROWTH_FACTOR_BINDING_PROTEINS_IGFBPS | 0.76 | 3.13 | 1.00E-03 | IGF2 IGFBP2 IGFBP6 |
| PID_LKB1_PATHWAY | -0.47 | -3.22 | 1.86E-03 | AKT1S1 BRSK1 BRSK2 CDC37 ESR1 MAP2 MAPT MARK2 MLST8 MTOR PRKAB1 PRKACA RPTOR SFN STK11 TP53 TSC1 TSC2 YWHAH YWHAZ |
| KEGG_PARKINSONS_DISEASE | -0.33 | -3.38 | 1.87E-03 | ATP5A1 ATP5D ATP5G3 CASP9 COX1 COX2 COX5B COX6A1 COX7C COX8A CYTB GPR37 HTRA2 ND1 ND2 NDUFA4 NDUFA5 NDUFAB1 NDUFB7 NDUFC1 NDUFS3 NDUFV1 NDUFV3 PARK7 PINK1 SDHC SDHD SLC25A31 UBA1 UQCR11 UQCRC1 UQCRQ |
| REACTOME_AXON_GUIDANCE | -0.25 | -3.85 | 1.92E-03 | ABLIM1 ABLIM3 AGRN AP2B1 ARHGAP35 ARHGEF11 ARHGEF12 CACNA1H CACNB1 CACNB2 CACNB3 CAP1 CDC42 CDK5R1 CNTN2 CNTNAP1 COL4A2 COL9A2 CRMP1 DLG3 DNM1 FES FGFR1 FYN HFE2 HSP90AB1 L1CAM LAMB1 LIMK2 MAP2K2 MAPK1 MAPK3 MYH10 MYL12B MYL9 NCAM1 NCK1 NRP2 NUMB PFN1 PIP5K1C PLCG1 PLXNA3 RAC1 RAF1 RHOG ROBO2 RPS6KA1 SCN1B SCN3B SCN4A SCN7A SCN8A SDCBP SEMA4D SEMA5A SEMA6A SIAH2 SLIT1 SLIT3 SPTB SPTBN1 SPTBN2 SPTBN4 SRC STIP1 TRPC6 UNC5A |
| REACTOME_DOWNREGULATION_OF_ERBB2_ERBB3_SIGNALING | -0.72 | -2.53 | 1.98E-03 | AKT1 NRG2 RNF41 UBA52 USP8 |
| REACTOME_NEURONAL_SYSTEM | -0.20 | -3.15 | 3.83E-03 | ADCY2 ADCY5 ALDH5A1 AP2B1 ARHGEF9 CACNA1B CACNB1 CACNB2 CACNB3 CAMK4 CAMKK1 CHAT CHRNA5 CHRNB2 CHRNB4 CHRND DLG3 EPB41L1 GABBR1 GABRA5 GJD2 GNB2 GNG3 GNG7 GNG8 GRIK5 GRIN2C GRIP2 HCN1 KCNA1 KCNB2 KCNC1 KCNC3 KCND1 KCND2 KCNH1 KCNH6 KCNJ10 KCNJ11 KCNJ6 KCNJ9 KCNK3 KCNK6 KCNMB4 MAPK1 NCALD NSF PANX1 PLCB2 PRKCG RAF1 RASGRF2 RPS6KA1 SLC17A7 SLC1A1 SLC32A1 STX2 STX5 STX8 STXBP1 SYN2 SYT2 VAMP1 |
| PID_PRLSIGNALINGEVENTSPATHWAY | -0.60 | -3.11 | 3.88E-03 | ATF5 BCAR1 CCNA2 CCNE1 CDK2 MAPK1 MAPK3 PTP4A3 RABGGTA RAC1 SRC |
| REACTOME_CDT1_ASSOCIATION_WITH_THE_CDC6_ORC_ORIGIN_COMPLEX | 0.41 | 3.13 | 3.91E-03 | MCM8 ORC4 ORC6 PSMA2 PSMB10 PSMB4 PSMB5 PSMB8 PSMB9 PSMC2 PSMC6 PSMD12 PSMD14 PSMD6 PSMD7 PSMD9 PSME1 PSMF1 |
| REACTOME_SIGNALING_BY_ERBB2 | -0.35 | -3.59 | 5.62E-03 | ADCY2 ADCY5 AKT1 AKT1S1 CAMK4 CASP9 CDC37 CDKN1A CDKN1B CUL5 ERBB4 FOXO4 FYN GSK3A HBEGF HRAS MAP2K1 MAP2K2 MAPK1 MAPK3 MATK MLST8 MTOR NRG2 PDE1A PDE1B PLCG1 PRKACA PRKACB PRKAR2A PRKCE PRKCG RAF1 RICTOR RNF41 SRC THEM4 TSC2 UBA52 USP8 |
| REACTOME_CALNEXIN_CALRETICULIN_CYCLE | -0.69 | -2.54 | 5.67E-03 | CALR CANX EDEM3 PRKCSH |
| REACTOME_NETRIN1_SIGNALING | -0.47 | -3.00 | 5.68E-03 | ABLIM3 CDC42 HFE2 NCK1 PLCG1 RAC1 SLIT1 SLIT3 SRC TRPC6 |
| PID_ILK_PATHWAY | -0.47 | -3.11 | 5.73E-03 | ACTN1 AKT1 AURKA CCND1 CDC37 CDC42 ELMO2 GIT2 ILK LIMS1 LIMS2 MYL9 PARP1 PARVB PPP1R14A PPP1R14B PPP1R14C PXN RAC1 RHOG RICTOR RUVBL1 RUVBL2 SNAI1 TNS1 ZEB1 ZYX |
| REACTOME_L1CAM_INTERACTIONS | -0.33 | -2.97 | 5.83E-03 | AP2B1 CNTN2 CNTNAP1 DLG3 DNM1 FGFR1 L1CAM LAMB1 MAP2K2 MAPK1 MAPK3 NCAM1 NRP2 NUMB RAC1 RPS6KA1 SCN1B SCN3B SCN4A SCN7A SCN8A SDCBP SPTB SPTBN1 SPTBN2 SPTBN4 SRC STIP1 |
| KEGG_GLYCEROLIPID_METABOLISM | -0.42 | -2.87 | 5.99E-03 | AGK AGPAT1 AGPAT4 CEL DGAT2 DGKD DGKI DGKZ GPAT2 LIPC PPAP2A |
| REACTOME_SYNTHESIS_OF_DNA | 0.33 | 3.12 | 6.01E-03 | CDC45 LIG1 MCM8 ORC4 ORC6 PCNA PSMA2 PSMB10 PSMB4 PSMB5 PSMB8 PSMB9 PSMC2 PSMC6 PSMD12 PSMD14 PSMD6 PSMD7 PSMD9 PSME1 PSMF1 RFC3 RFC4 RPA1 |
| KEGG_GLYCEROPHOSPHOLIPID_METABOLISM | -0.36 | -3.00 | 7.55E-03 | AGPAT1 AGPAT4 AGPAT6 CDIPT CHAT DGKA DGKB DGKD DGKG DGKI DGKZ GPAM GPAT2 GPD1 LPCAT1 LPCAT3 LPCAT4 LYPLA2 PCYT2 PGS1 PLA2G1B PLA2G2A PLA2G2C PLA2G3 PLA2G6 PLD1 PPAP2A PPAP2C PTDSS1 TAZ |
| KEGG_PEROXISOME | 0.37 | 3.35 | 7.95E-03 | ABCD2 ACOX3 ACSL3 ECI2 EPHX2 FAR1 GSTK1 HMGCL HSD17B4 IDH2 NUDT12 PEX2 PEX26 PHYH PXMP2 SLC25A17 SLC27A2 SOD2 |
| REACTOME_RNA_POL_I_TRANSCRIPTION_TERMINATION | 0.62 | 2.81 | 8.39E-03 | CCNH CDK7 GTF2H2 MNAT1 POLR1B TAF1B UBTF |
| REACTOME_INTERFERON_SIGNALING | 0.31 | 3.49 | 8.62E-03 | CIITA EIF2AK2 FLNB HLA-DQA1 IFI27 IFI35 IFIT2 IFITM1 IRF6 IRF7 IRF9 ISG15 JAK2 KPNA1 KPNA4 KPNB1 MX1 NUP153 NUP155 NUP35 OAS1 PPM1B PSMB8 PTPN2 SOCS3 TRIM25 UBE2L6 USP18 VCAM1 |
| REACTOME_MITOTIC_G1_G1_S_PHASES | 0.28 | 3.17 | 9.78E-03 | CCNH CDC45 CDK7 CDKN2C E2F5 LIN52 LIN54 LIN9 MCM8 MNAT1 ORC4 ORC6 PCNA PSMA2 PSMB10 PSMB4 PSMB5 PSMB8 PSMB9 PSMC2 PSMC6 PSMD12 PSMD14 PSMD6 PSMD7 PSMD9 PSME1 PSMF1 RBL1 RPA1 TK2 |
| MSigDB Pathway | ES | NES | p-value | genes in leading edge |
